# BID-F1 and BID-F2 Domains of *Bartonella henselae* Effector Protein BepF Trigger Together with BepC the Formation of Invasome Structures

**DOI:** 10.1371/journal.pone.0025106

**Published:** 2011-10-17

**Authors:** Matthias C. Truttmann, Patrick Guye, Christoph Dehio

**Affiliations:** 1 Focal Area Infection Biology, Biozentrum of the University of Basel, Basel, Switzerland; 2 Department of Biological Engineering, Massachusetts Institute of Technology (MIT), Cambridge, Massachusetts, United States of America; Indian Institute of Science, India

## Abstract

The gram-negative, zoonotic pathogen *Bartonella henselae* (*Bhe*) translocates seven distinct *Bartonella* effector proteins (Beps) via the VirB/VirD4 type IV secretion system (T4SS) into human cells, thereby interfering with host cell signaling [1], [2]. In particular, the effector protein BepG alone or the combination of effector proteins BepC and BepF trigger massive F-actin rearrangements that lead to the establishment of invasome structures eventually resulting in the internalization of entire *Bhe* aggregates [2], [3]. In this report, we investigate the molecular function of the effector protein BepF in the eukaryotic host cell. We show that the N-terminal [E/T]PLYAT tyrosine phosphorylation motifs of BepF get phosphorylated upon translocation but do not contribute to invasome-mediated *Bhe* uptake. In contrast, we found that two of the three BID domains of BepF are capable to trigger invasome formation together with BepC, while a mutation of the WxxxE motif of the BID-F1 domain inhibited its ability to contribute to the formation of invasome structures. Next, we show that BepF function during invasome formation can be replaced by the over-expression of constitutive-active Rho GTPases Rac1 or Cdc42. Finally we demonstrate that BID-F1 and BID-F2 domains promote the formation of filopodia-like extensions in NIH 3T3 and HeLa cells as well as membrane protrusions in HeLa cells, suggesting a role for BepF in Rac1 and Cdc42 activation during the process of invasome formation.

## Introduction

*Bartonella henselae* (*Bhe*) is a worldwide distributed, zoonotic pathogen. In its feline reservoir host, it causes an asymptomatic, intraerythrocytic bacteraemia [4]. Accidental transmission of *Bhe* from cats to humans can manifest in a variety of clinical symptoms, ranging from the so-called cat-scratch disease in immuno-competent patients to bacillary angiomatosis or peliosis in immuno-compromised persons, respectively [5].

*Bhe* expresses a VirB/VirD4 type IV secretion system (T4SS) that mediates translocation of the *Bartonella* effector proteins (Beps) BepA to BepG into the host cell cytosol [1], [6]. The Bep effectors share a common basal architecture, consisting of an N-terminal effector domain and a bi-partite translocation signal composed of at least one BID domain (*Bartonella* intracellular delivery) and a positively charged C-terminus [7], [8]. Effectors BepA, BepB and BepC all contain a single FIC domain in proximity to their respective N-terminus, while BepD, BepE and BepF display tyrosine/proline-rich repeats in their N-terminal portion [7], [8]. Interestingly, effectors BepE, BepF and BepG all contain multiple BID domains while BepG consists exclusively of four BID domains flanked by short linker regions [2], [8], [9].

Bep translocation into the host cell promotes a variety of distinct phenotypes that include: (i) inhibition of apoptosis, (ii) activation of the pro-inflammatory response, (iii) capillary-like sprout formation of endothelial cell aggregates and (iv) host cell invasion by a cellular structure named the invasome [2], [6], [8], [10], [11]. *Bhe* internalization via the invasome route is a well controlled multi-step process, consisting of *Bhe* adherence to the cell surface, *Bhe* aggregation, *Bhe* engulfment by plasma-membrane-derived membrane protrusions and eventually *Bhe* internalization [12]. Invasome formation can be triggered in a redundant manner, either by BepG alone or by the combined action of effectors BepC and BepF [2], [3].

Various pathogenic bacteria translocate effector proteins into their respective host cells that interfere with Rho GTPase signaling events [13], [14]. Rho GTPases interact in their GTP-bound form with multiple downstream proteins, thereby transmitting incoming signals to basal levels. In contrast, GDP-bound GTPases are not able to bind to and activate their interaction partners [15]. GTPase signaling is in general controlled by GAP, GEF and GDI proteins. While GAPs (GTPase-activating proteins) stimulate the turn-over of the GTP to GDP, GEF (guanine nucleotide exchange factor) increase the exchange rate of GDP with GTP. GDI (guanine nucleotide dissociation inhibitor) bind to the C-terminal lipid groups of GTPases, thereby preventing membrane binding and stabilizing them in the inactive state in the cytosol [15], [16]. Pathogenic bacteria translocate various GAPs or GEFs into the host cell in order to subvert Rho GTPase signaling: In example, *Salmonella enterica* effector SptP or *Yersinia enterocolitica* effector YopE act as GAPs of Rho GTPases, while the *S. enterica* protein, SopE as well as *Escherichia coli* effector MAP posses GEF functionality on Rho-family GTPases [13], [14]. Recently, a new family of bacterial effector proteins sharing a common Trp-xxx-Glu motif (WxxxE motif) was shown to interfere with Rho GTPase signaling [13], [17]. These WxxxE-family proteins, later shown to be Rho GEFs, include SifA and SifB from *Salmonella*, MAP and EspM/M2 from *E.coli* as well as IpgB2 and IpgB1 from *Shigella* [13], [17], [18]. The WxxxE motif was demonstrated to be essential for GEF function although it is not directly involved in establishing contact with the target Rho GTPases [18]. Alternatively to exhibit GAP or GEF functions, bacterial effector proteins were shown to directly interfere with Rho GTPase signaling by promoting chemical modifications of GTPases (ADP-rybosylation, glucosylation, AMPylation) [19], [20], [21], [22] or indirectly by interacting with Rho GTPase regulators such as Dock180, Crk or ELMO [19], [20], [21], [22].

In this study, we investigate the function of the *Bartonella* effector protein BepF. We show that the isolated BID-F1 or BID-F2 domains - together with BepC - are sufficient to trigger invasome establishment. Further, we demonstrate that constitutive-active Cdc42 or Rac1 can substitute for BepF in the BepC/BepF-dependent invasome formation pathway, suggesting a regulatory role of BepF on the small Rho GTPases during the process of invasome formation.

## Materials and Methods

### Bacterial Strains, Growth Conditions, Conjugations

*Bhe* strains were cultured as previously described on solid agar plates (Columbia base agar supplemented with 5% sheep blood and appropriate antibiotics). *E. coli* strains were grown on solid agar plates (Luria Bertani broth) supplemented with appropriate antibiotics. Triparental matings between *E. coli* and *Bhe* strains were performed as described [23]. Table S1 lists all bacteria strains used in this study.

### Plasmid Construction

DNA manipulations were carried out following standard protocols. Vectors pCD353, pMS007, pPG100 and derivatives, pRS79, pMT563 and pTR1769 as well as peGFP-Cdc42, peGFP-Cdc42, pRK5mycL61-Cdc42, pRK5mycL61-Rac1 have been described before (see table S1 for plasmid origins). eGFP-Bep fusion plasmids pMT560, pMT562, pMT567, pMT591, pMT592, pMT593, pMT597. pMT612, pMT613 and pMT614 were obtained by PCR amplification of the respective insert with the corresponding primers, cutting the purified PCR products with XmaI and XbaI and their ligation into pWAY21 (eGFP, Molecular Motion, Montana Labs) cut accordingly. pMT001, pMT004, pMT005, pMT030, pMT031 and pMT52 were generated by PCR amplification of the respective insert with the corresponding primers, cutting the purified PCR products with NdeI and their ligation into NdeI-digested pPG100. All constructs were sequence confirmed. Tables S1 and S2 list all plasmids and primers constructed or used in this study.

### Cell Lines and Cell Culture

HeLa Kyoto β cells [24] and NIH3T3 cells [25] were cultured in DMEM (Gifco, invitrogen) supplemented with 10% fetal calf serum (FCS).

### Transfection and Infection Assays

Transfection and infection of HeLa cells was performed as described [3]. In brief, 4500 cells were seeded into a well of a 96-well plate, and after over-night incubation transfected with DNA using Lipofectamine2000 (invitogen), following manufacturer's instructions. Cells were washed once with phosphate-buffered saline (PBS) and supplemented with fresh DMEM/10%FCS medium 6–8 h post transfection. Cells were further incubated for 24 h at 35°C, 5% CO_2_ before continuing with the respective assays.

HeLa infections were carried out as described [3]. In brief, HeLa cells were infected with *Bhe* at a multiplicity of infection (MOI) = 500 per strain in 100 µl medium M199/10%FCS supplemented with 500 µM IPTG (Promega). Following 48 h incubation cells were fixed with para-formaldehyde (PFA).

Transfection of NIH 3T3 cells was performed following manufacturer's instructions. Briefly, cells were seeded out at a density of 30000/well of a 24 well plate and incubated over night. The next day, 200 µl optimem was mixed with 2 µg of plasmid DNA and 6 µl of lipofectamine2000 and incubated for 30 min. Afterwards, 100 µl of the transfection mix was added to the cells together with 400 µl of fresh DMEM/10%FCS and incubated for 4 h. Then, medium was exchanged with 500 µl fresh DMEM/10%FCS and cells were incubated for 48 h at 35°C, 5% CO_2_.

### Immunoprecipitation (IP) and Immunoblot analysis

IP was performed as described elsewhere [11]. Expression of novel N-terminal FLAG-tagged and NLS-Cre-Bep fusion proteins was verified by analysis of total *Bhe* lysates obtained from *Bhe* grown on CBA plates containing 500 µM IPTG. Proteins were run on a SDS-PAGE gel for separation and transferred onto nitrocellulose membranes (Hybond, Amersham Biosciences) and probed against the FLAG epitope using mouse monoclonal anti-FLAG antibody M2 (Sigma, 1∶1000). Novel eGFP-Bep fusion proteins were assessed for their stability by analysis of total cell lysates obtained from HeLa cells transfected with plasmids encoding the respective constructs and incubated for 24 h. After protein separation by SDS-PAGE and transfer onto nitrocellulose, membranes were examined for the presence of eGFP using rabbit monoclonal anti-GFP antibody (Molecular Probes, 1∶5000). In all experiments, secondary horseradish peroxidase-conjugated antibody (Amersham, 1∶10000) was visualized by enhanced chemiluminescence (PerkinElmer).

### Immunofluorescent (IF) labeling

Indirect IF labeling was performed as described [12]. Standard 96-well plate assays were stained with TRITC-phalloidin (Sigma, 100 µg/ml stock solution, final concentration 1∶400), and DAPI (Roche, 0.1 mg/ml) using a Tecan Eoware freedom pipeting robot. Glasslides for confocal microscopy were stained with Cy5-phalloidine (Sigma, 100 µg/ml stock solution, final concentration 1∶100), and DAPI.

### Semi-automatic image analysis, invasome quantification and microscopy

Image analysis and invasome quantification was performed as described [3]. In brief, cells were automatically imaged in up to three different wavelengths depending on the applied cell staining. The number of cells per image was determined by MetaExpress in-build analysis modules (CountNuclei) and invasomes on the very same images were defined and counted by eye. In every experiment, at least 500 cells were analyzed per condition.

### Epi-fluorescence and Confocal Laser Scanning Microscopy

Epi-fluorescence and confocal Laser Scanning was performed exactly as described earlier [3].

In brief, 96-well plates were imaged with an ImagXpress Micro (IXM) automated microscope (Molecular devices). For confocal laser microscopy, specimens were visulaized using an IQ iXON spinning disc system (Andor) in combination with an IX2-UCB microscope (Olympus). Images were exported and finalized using Metamorph, ImageJ and Adobe Photoshop.

### Scanning electron microscopy (SEM)

SEM analysis was performed exactly as described before [3]. In brief, cells were seeded onto glass slides and treated as described above (infection and transfection assays). Following incubation, probes were washed and fixed with 250 µl of 2.5% glutaraldehyde for 30 min at RT. Afterwards, cells were washed twice and the samples were subsequent dehydrated with an ethanol step gradient (30%, 50%, 70%, 90%, 100%; 15 min each) at 4°C. Thereafter, samples were critical point-dried and sputter-coated with a 3 nm thick Platin layer. Images were taken on a Hitachi S-4800 field emission scanning electron microscope, using an acceleration voltage of 2 kV.

## Results

### BepF tyrosine phosphorylation is not required for invasome formation

**Figure 1:**
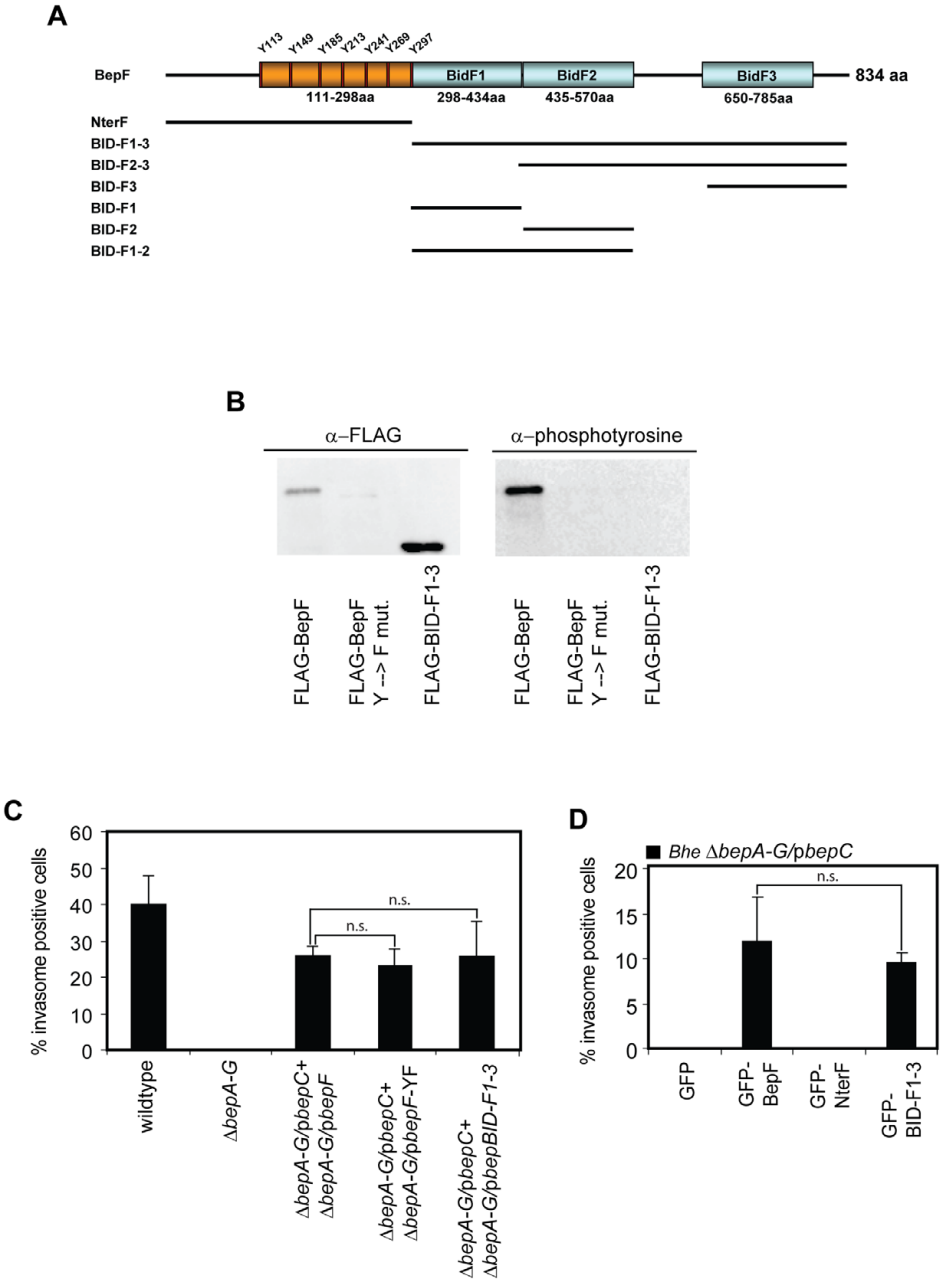
Tyrosine phosphorylation of BepF is not essential for invasome formation. (**A**) Schematic representation of BepF, the tyrosine phosphorylation sites and the individual domains. The black bars indicate the corresponding regions represented by the GFP- or FLAG-tagged BepF truncated constructs used in this study. (**B**) HeLa cells were infected with indicated *Bhe* strains at an MOI = 500 for 48 h. Following anti FLAG-IP, samples were subjected to SDS-PAGE, transferred onto a nitrocellulose membrane and probed using anti-FLAG antibodies (left panel). Upon stripping, membranes were re-probed using anti-phosphotyrosine antibodies (right panel). (**C**) HeLa cells were infected with indicated *Bhe* strains at an MOI = 500 for 48 h. Following fixation, staining with TRITC-Phalloidin and DAPI and image acquisition by automated epifluorescence microscopy, invasomes were quantified (n>500 cells). Results of at least three independent experiments +/− standard deviation are depicted. Student's t-test was performed as indicated. (**D**) HeLa cells were transfected with the indicated plasmids for 24 h and thereafter infected with *Bhe ΔbepA-G*/p*BepC* at an MOI = 500 for 48 h. Following fixation, staining with TRITC-Phalloidin and DAPI and image acquisition by automated epifluorescence microscopy, invasomes were quantified (n>500 cells). Results of at least three independent experiments +/− standard deviation are depicted. Student's t-test was performed as indicated.

In previous work, we have shown that BepC together with BepF can trigger invasome formation [3]. However, the molecular details of the function of either of the two proteins remained to be determined. *In silico* analysis of the sequence of BepF revealed that BepF contains a tyrosine-rich repeat motif close to its N-terminus, which is linked to three BID domains. The first and the second BID domain, BID-F1 and BID-F2, are fused together while the third BID domain, BID-F3, is linked via a short spacer sequence to BID-F2 (Fig. 1A). Web-based sequence analysis of BepF using Scansite [26] (http://scansite.mit.edu/) and NetPhos [27] (http://www.cbs.dtu.dk/services/NetPhos/) yielded in high probability predictions of multiple tyrosine phosphorylations of the tyrosine-rich motifs [E/T]PLYAT (fig, S1A, B, C). Furthermore, previous work demonstrated that short, synthesized peptide fragments containing the [E/T]PLYAT motif of BepF are *in vitro* phosphorylated and interact with Crk, RasGAP and Grb2 [11]. To check whether the tyrosine-rich repeats of BepF are indeed phosphorylated upon host cell entry and contribute to invasome formation, we generated two BepF mutants, one having all seven tyrosine replaced with phenylalanine (further referred to as BepF-YF) and one mutant consisting only of the three BIDF domains and the positively charged C-tail (further referred to as BID-F1-3) (Fig. 1A). HeLa cells were thereafter co-infected with the effector-deficient *Bhe* strain *ΔbepA-G* expressing FLAG-tagged BepC and *Bhe ΔbepA-G* strains expressing BepF or BepF mutant constructs BepF-YF, BID-F1-3 with an MOI = 500 per strain for 48 h. The stability of FLAG-tagged mutant constructs of BepF was verified by Western blotting (Fig. S2A). Following immunoprecipitation using anti-FLAG agarose beads, tyrosine phosphorylation was analyzed by Western blotting. The results clearly showed that wild-type BepF is tyrosine phosphorylated in the host cell, while neither of the two mutant constructs displayed any detectable tyrosine phosphorylation signal (Fig. 1B), indicating that the N-terminal tyrosine-containing repeat motifs are indeed phosphorylated in the host cell. Next, we investigated if the tyrosine-rich repeat is required for BepF to contribute to invasome-mediated *Bhe* internalization. Therefore, we infected HeLa cells with *Bhe* wild-type, *Bhe ΔbepA-G* or combinations of *Bhe ΔbepA-G*/p*BepC* and *Bhe ΔbepA-G*/p*bepF*, *ΔbepA-G*/p*bepF-YF* or *ΔbepA-G*/p*BID-F1-3* (Fig. 1C). Quantification of invasome formation of fixed, stained and microscopically imaged cells demonstrated that BID-F1-3 was sufficient to trigger invasome formation together with BepC to the same level as wild-type BepF or BepF-YF. To further strengthen that point, we generated eGFP-tagged fusion proteins containing either only the N-terminal part of BepF (NterF) or the BID-F1-3 region (Fig. 1A, 1D). HeLa cells were transfected with plasmids encoding for eGFP, eGFP-BepF, eGFP-NterF and eGFP-BID-F1-3 and, after 24 h incubation, infected with *Bhe ΔbepA-G*/p*bepC* at an MOI = 500 for another 48 h. Stable expression of the eGFP-fusion was verified by Western blotting (Fig. S2B). The obtained data were in line with our previous finding: HeLa cells ectopically expressing either eGFP-BepF or eGFP-BID-F1-3 and infected with *Bhe ΔbepA-G*/p*bepC* showed invasome formation at a frequency of about 10%, while HeLa cells expressing GFP-NterF and infected with the same strain did not show any invasomes. Taken together, we show that the BID domains BID-F1-3 are sufficient to trigger invasome formation together with BepC. Further, we show that, although tyrosine-phosphorylated in the host cell, the N-terminal tyrosine-containing repeat motif of BepF does not contribute to BepC/BepF-dependent invasome formation.

### The BID domains BID-F1 and BID-F2 of BepF together with BepC are sufficient to promote invasome formation

**Figure 2:**
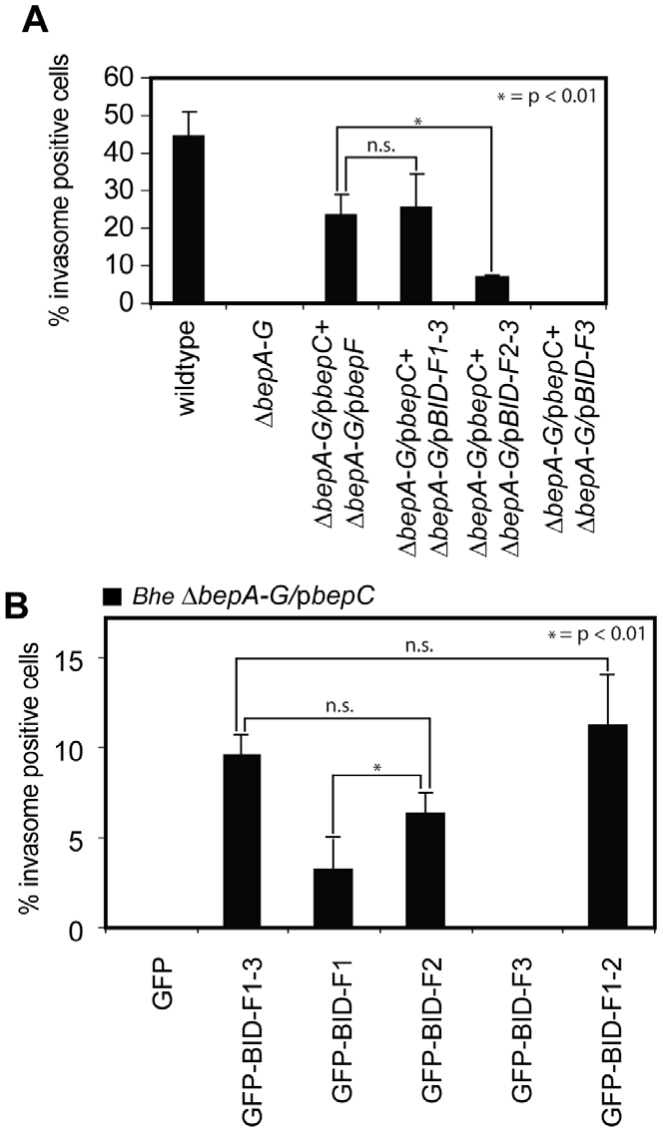
BID-F1 and BID-F2 are sufficient to trigger invasome formation together with BepC. (**A**) HeLa cells were infected with indicated *Bhe* strains at an MOI = 500 for 48 h. Following fixation, staining with TRITC-Phalloidin and DAPI and image acquisition by automated epifluorescence microscopy, invasomes were quantified (n>500 cells). Results of at least three independent experiments +/− standard deviation are depicted. Student's t-test was performed as indicated. (**B**) HeLa cells were transfected with indicated plasmids for 24 h and thereafter infected with *Bhe ΔbepA-G*/p*BepC* at an MOI = 500 for 48 h. Following fixation, staining with TRITC-Phalloidin and DAPI and image acquisition by automated epifluorescence microscopy, invasomes were quantified (n>500 cells). Results of at least three independent experiments +/− standard deviation are depicted.

In a next step, we tested whether individual BID domains of BepF could contribute to invasome formation in combination with BepC. Therefore, we first cloned FLAG-tagged BepF mutant constructs that consist of BID-F2-3 or BID-F3 and transformed the plasmids into *Bhe ΔbepA-G* (Fig. 1A). Fusion construct expression and stability was tested by Western blotting (Fig. S2A). *Bhe* strains *ΔbepA-G*/p*BID-F2-3 and ΔbepA-G*/p*BID-F3* were tested in co-infection experiments with *Bhe ΔbepA-G*/p*BepC* according to the standard protocol. Quantification of invasome formation on fixed, stained and imaged cells indicated that the removal of the first BID domain (BID-F1) reduced invasome formation by about 70% compared to BID-F1-3, while the removal of both BID-F1 and BID-F2 together lead to a complete abolishment of invasome formation (Fig. 2A). To investigate the capacity of BID-F1 and BID-F2 to contribute to invasome formation in more details, we generated plasmids encoding for eGFP-tagged constructs eGFP-BID-F1, eGFP-BID-F2, eGFP-BID-F3 and eGFP-BID-F1-2. Fusion protein stability was verified by Western blotting (Fig. S2B). Following transfection of HeLa cells with the indicated constructs, cells were infected with *Bhe ΔbepA-G*/p*bepC* for 48 h. The results showed that both BID-F1 and BID-F2 together with BepC are able to promote invasome formation while it was absent from cells expressing eGFP-BID-F3 and infected with *Bhe ΔbepA-G*/p*bepC* (Fig. 2B). Interestingly, eGFP-BID-F2 was significantly more potent than eGFP-BID-F1 to promote invasome establishment and eGFP-BID-F1-F2 was promoting invasome formation to the same extent than BID-F1-3, each in combination with BepC. In summary, our results show that BID-F1 and BID-F2, but not BID-F3 domains are individually sufficient to mediate invasome formation in combination with BepC.

### Disruption of the WxxxE motif in BID-F1 interferes with BID-F1 function

**Figure 3:**
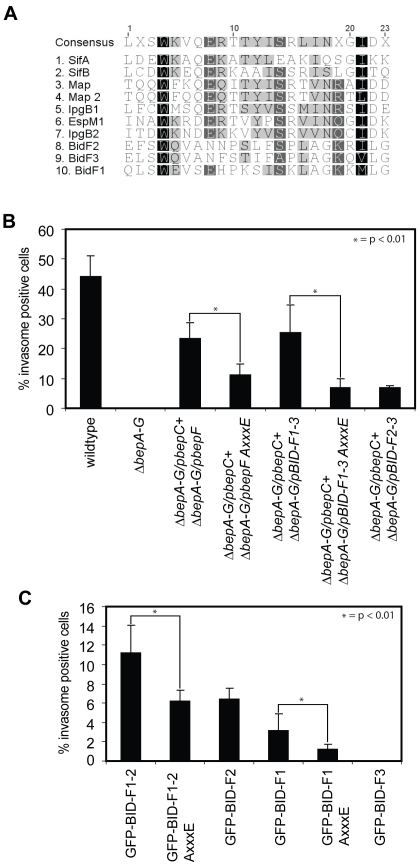
Disruption of the WxxxE motif in BID-F1 interferes with BID-F1 function during invasome formation. (**A**) Amino acid sequence alignment of described WxxxE effectors and BepF domains BID-F1, BID-F2 and BID-F3. Conserved amino acids are highlighted in grey to dark depending on the level of conservation. (**B**) HeLa cells were infected with indicated *Bhe* strains at an MOI = 500 for 48 h. Following fixation, staining with TRITC-Phalloidin and DAPI and image acquisition by automated epifluorescence microscopy, invasomes were quantified (n>500 cells). Results of at least three independent experiments +/− standard deviation are depicted. Student's t-test was performed as indicated. (**C**) HeLa cells were transfected with indicated plasmids for 24 h and thereafter infected with *Bhe ΔbepA-G/*p*BepC* at an MOI = 500 for 48 h. Following fixation, staining with TRITC-Phalloidin and DAPI and image acquisition by automated epifluorescence microscopy, invasomes were quantified (n>500 cells). Results of at least three independent experiments +/− standard deviation are depicted. Student's t-test was performed as indicated.

In 2008, Alto *et al* proposed a family of bacterial effector proteins containing a WxxxE motif to be mimics of host cell GTPases [17]. This statement was later revised and it was shown for multiple instances that translocated bacterial proteins containing the WxxxE motif act as GEFs for Rho family GTPases [28]. Sequence analysis of the BIDF domains showed that BID-F1 contains a WxxxE motif as well, while BID-F2 and BID-F3 harbor a closely related motif at the same position, WxxxN. However, amino acid sequence alignments of BID-F1, BID-F2 and BID-F3 with known WxxxE-family GEFs showed low sequence conservation besides the motif itself (Fig. 3A). Nevertheless, we decided to further focus on BID-F1, since it contains an intact WxxxE motif, and mutated tryptophan-362 into alanine in various BepF-related constructs to disrupt the WxxxE motif (AxxxE). Thereafter, we co-infected HeLa cells according to the standard protocol with *Bhe ΔbepA-G*/p*bepC* and *ΔbepA-G*/p*bepF* W362A, *ΔbepA-G*/p*BID-F1-3* W362A or *ΔbepA-G*/p*BID-F2-3* and checked for invasome formation. Mutant protein stability was tested by Western blotting (Fig. S2A). The obtained results demonstrate that, upon changing the WxxxE motif to AxxxE, the capacity of BepF as well as BID-F1-3 to contribute to invasome formation decreased to the level obtained for co-infections with *ΔbepA-G*/p*bepC* and *ΔbepA-G*/p*BID-F2-3*, thus basically eradicating the contribution of BID-F1 to the process of invasome formation (Fig. 3B). Next, we introduced the mutation into our eGFP-fusion constructs and quantified invasome formation on HeLa cells ectopically expressing eGFP-fusion proteins and infected with *Bhe ΔbepA-G*/p*BepC* following standard protocols. GFP-fusion protein stability was tested by Western blotting (Fig. S2B). These results were in line with our previous findings: the introduced W362A mutation in eGFP-BID-F1-2 decreased invasome formation down to the level found for eGFP-BID-F2 alone in combination with BepC. Furthermore, mutating the WxxxE motif in eGFP-BID-F1 significantly decreased invasome formation compared to wild-type eGFP-BID-F1. Comparing the amino acid sequences of BIDF domains with characterized WxxxE-family GEF proteins, we identified a conserved serine residue located six amino acids downstream of the glutamic acid of the WxxxE motif (Fig. 3A). This serine was present in all WxxxE-family proteins except for SifA, while being present in BID-F2 but absent in BID-F3. To test whether this serine residue may play a role in BID-F1 and BDF2 functionality during invasome formation, we constructed mutant constructs encoding for GFP-BID-F1 S372A, GFP-BID-F1 W362A/S372A and GFP-BID-F2 S508A. The constructs were tested in standard transfection-infection assays and invasome formation was quantified after 48 h of infection with *Bhe ΔbepA-G*/p*BepC*. The results showed that mutation of serines 372 and 508 did not affect invasome formation, implying that the indicated residue is not critical to maintain BID-F1 and BID-F2 domain function and structure (Fig. S3).

Concluding, our results indicate that the WxxxE motif found in BID-F1 is essential for the function of the BID-F1 domain and that the conserved serine residue downstream of the WxxxE motif is not critical to maintain BID-F1 and BID-F2 functionality.

### BepF can be substituted by expression of constitutive active Cdc42 or Rac1 during BepC/BepF-dependent invasome formation

**Figure 4:**
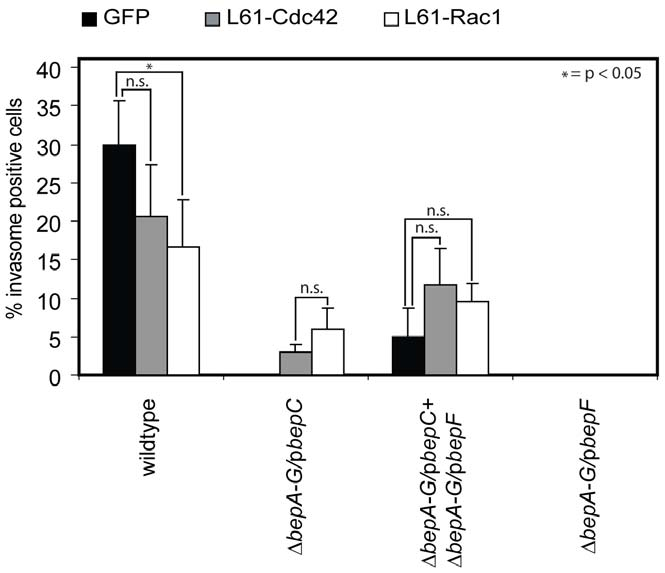
L61-Cdc42 and L61-Rac1 can substitute for BepF in the process of invasome formation. HeLa cells were transfected with indicated plasmids for 24 h and thereafter infected with *Bhe ΔbepA-G*/p*BepC* at an MOI = 500 for 48 h. Following fixation, staining with TRITC-Phalloidin and DAPI and image acquisition by automated epifluorescence microscopy, invasomes were quantified (n>500 cells). Results of at least three independent experiments +/− standard deviation are depicted. Student's t-test was performed as indicated.

Several bacterial effectors containing the WxxxE motif were shown to act as GEFs for the small GTPases RhoA, Rac1 and Cdc42 [13]. Previous work on *Bhe*-triggered invasome formation has further demonstrated that Cdc42 and Rac1, but not RhoA, are required for invasome formation [2], [3]. To test whether BepF interferes with Rac1- or CdC42-mediated signaling, we transfected HeLa cells with plasmids encoding for myc-tagged constitutive active Cdc42 (L61-Cdc42) or Rac1 (L61-Rac1). After 24 h of incubation, cells were infected with *Bhe ΔbepA-G*/p*BepC* at an MOI = 500 and incubated for another 48 h. Following fixation and staining, invasome formation was quantified (Fig. 4). Our results showed that *Bhe ΔbepA-G*/p*BepC* could indeed promote invasome formation on HeLa cells expressing either L61-Rac1 or L61-Cdc42. Further, we also observed a more than 50% increase in invasome frequency on HeLa cells expressing either constitutive active GTPase and infected with *Bhe ΔbepA-G*/p*BepC* and *ΔbepA-G*/p*bepF* compared to empty vector transfected cells. Interestingly, invasome formation on HeLa cells expressing L61-Cdc42 or L61-Rac1 and infected with *Bhe* wild-type decreased compared to the empty vector control, thereby confirming previous published results [2]. The fact that substitution of BepF with L61-Cdc42 or L61-Rac1 leads to significantly less invasome formation as the combined action of BepC/BepF indicates that the activity of Cdc42 and Rac1 is essential for certain steps of invasome establishment but may act rather inhibitory on other aspects of the entire process.

### BepF triggers the formation of filopodia-like extensions and membrane protrusions on HeLa and NIH 3T3 cells

**Figure 5:**
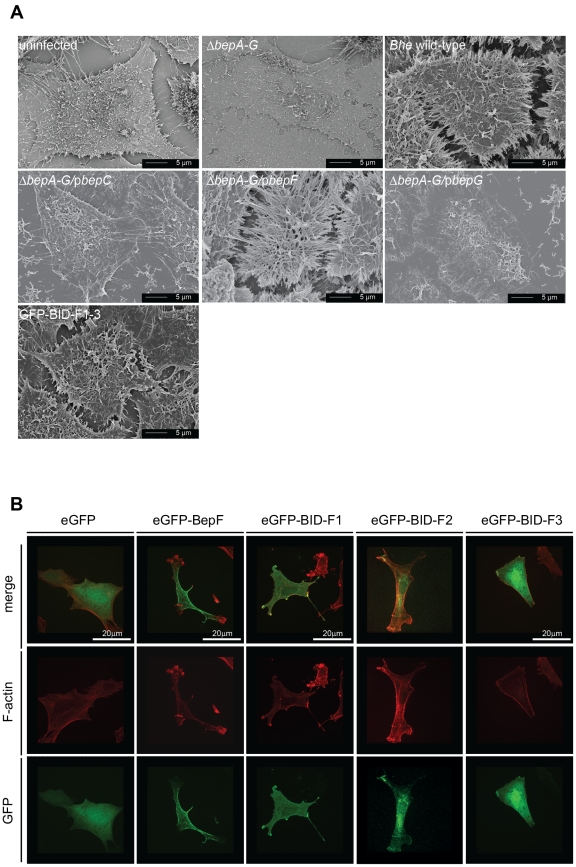
BepF triggers the formation of filopodia-like structures. (**A**) HeLa cells were infected with the indicated *Bhe* strains at an MOI = 500 for 48 h. Following fixation, and critical-point drying, cells were visualized by TEM microscopy. Representative images of parallel infections are depicted. Scale bare is indicated. (**B**) Swiss 3T3 cells were serum-starved for 48 h and thereafter transfected with indicated plasmids for 24 h. Following fixation, and staining with TRITC-Phalloidin and DAPI, cells were visualized by confocal microscopy. Representative images of parallel transfections are depicted. Scale bars are indicated.

Although BepF has been shown to infrequently trigger the formation of small actin foci, the function of BepF has mainly been investigated in the context of invasome formation [3]. Based on the finding that the constitutive active GTPases L61-Cdc42 and L61-Rac1 can substitute for BepF function we tested for a BepF-specific phenotype on the F-actin cytoskeleton level that is related to the action of L61-Cdc42 or L61-Rac1. To this end, we infected HeLa cells with various *Bhe* strains at a high MOI (1000) for 48 h to trigger maximal phenotypic penetrance. As previously reported host cell viability was unaffected under these infection conditions [3]. After cell fixation, we analyzed the cells by scanning electron microscopy (SEM). Uninfected as well as *Bhe ΔbepA-G*, *ΔbepA-G*/p*BepC* or *ΔbepA-G*/p*bepG* infected HeLa cells showed low levels of filapodia-like structures or membrane protrusions. (Fig. 5A). In contrast, HeLa cells infected with *Bhe* wild-type or *Bhe ΔbepA-G*/p*BepF* displayed drastically changed cell morphology and showed massive formation of filopodia-like structures as well as membrane protrusions that frequently contacted neighboring cells. The previously reported small actin foci promoted by BepF on HUVECs were completely absent on HeLa cells [3].

In a next step, we tested our eGFP-BIDF fusion constructs in the same TEM-based assay. We found that BID-F1 as well as BID-F2, but not BID-F3 or BID-F1 AxxxE induced the formation of filopodial extensions and membrane protrusions (). To strengthen our findings, we repeated the experiments with the eGFP-fusion constructs in NIH 3T3 cells, a cellline well known for a highly responsive actin cytoskeleton that is often used to study stress fibers, lamelipodia and filopodia formation upon system perturbation [29]. To this end, we transfected NIH 3T3 cells with indicated plasmids encoding for eGFP-fusion constructs as well as proper controls. After fixation and staining, cells we analyzed the actin cytoskeleton phenotype of GFP-positive cells. The results showed that eGFP-tagged full-length BepF, BID-F1 and BID-F2 induced a change in actin cytoskeleton morphology that is phenotypically comparable to the expression of L61-Rac1 or L61-Cdc42 in these cells while neither eGFP control, eGFP-tagged BID-F3 nor eGFP-tagged BID-F1 AxxxE fusion proteins affected the F-actin organization of NIH 3T3 cells (Fig. 5B, S5). In summary, our data suggests that the BepF domains BID-F1 and BID-F2 are involved in the regulation, in particular the activation of Rac1 and Cdc42.

## Discussion

The *Bartonella henselae* effector protein BepF has previously been implicated in triggering invasome formation together with BepC in a cofilin1-dependent manner [3]. Here, we show that the individual BID domains BID-F1 and BID-F2, but not BID-F3 are sufficient to promote invasome formation together with BepC. Sequence analysis of the three BepF BID domains implies that BID-F2 and BID-F3 are more homologue to each other than to BID-F1; however, the general level of sequence homology is low. Thus, from sequence comparison it is not evident why BID-F1 and BID-F2 can contribute to invasome formation while BID-F3 cannot.

Besides the three BID domains, BepF contains a tyrosine-rich repeat motif that is phosphorylated in the host cell upon effector translocation. Interestingly, the replacement of all tyrosine residues as well as the complete removal of that protein portion did not interfere with BepC/BepF-mediated invasome formation, nor with BepF triggered formation of filopodial cell extensions. It is tempting to assume that BepF may interact with multiple SH2-domain containing proteins that can bind to the phosphor-tyrosine scaffold of BepF. However, we were so far unable to identify a cellular phenotype that is linked to the N-terminal portion of this translocated effector protein.

The interference with Rho GTPases to subvert host signaling cascades is a frequent function associated with translocated bacterial effector proteins. Several distinct mechanisms have been reported yet, including bacterial GEF and GAP proteins (SopE, SptP) [13], covalent modification of the target GTPases by AMPylation (VopS, IbpA) [30], glucosylation (TcdA/B) [19] or ADP-rybosylation (C3) [31] as well as the deamidation (CNF1) [20] and partial proteolytic degradation (YopT) [32] of Rho-family G proteins. In this report, we show that BepF can be replaced by constitutive-active CDC42 or Rac1 in the process of invasome formation. The findings that neither constitutive active GTPase was as potent as BepF to contribute to invasome formation and that over-expression of both constitutive active GTPases interfered with BepC/BepF- or *Bhe* wild-type promoted invasome assembly suggests that the tempo-spatial control of Cdc42 and Rac1 activity is important for the establishment of invasome structures. This hypothesis is in accordance with the published data on invasome formation, which showed that the assembly of the massive actin structure is followed by the eventual retraction of the actin arrangement that leads to the release of the bacteria into the host cell [12]. The constitutive activation of Cdc42 and Rac1 that both control processes associated with F-actin filament elongation and cell protrusion formation may be central for the assembly of the invasome structure but rather disadvantageous for the retraction and the disassembly thereof. A BepF-dependent activation of Cdc42 and Rac1 is further indicated the BepF-triggered formation of filopodia-like cell extensions and membrane protrusions on HeLa and NIH 3T3 cells. [33].

Recent work on translocated bacterial WxxxE GEF proteins suggested that the motif itself may have mainly structural roles, in particular by maintaining the conformation of the putative catalytic loop through hydrophobic contacts with surrounding residues [18]. As BID-F1 contains an intact WxxxE motif and its disruption interferes with BID-F1 function, it is tempting to speculate that BepF is a further WxxxE-family bacterial GEF protein. However, sequence alignments of the distinct WxxxE-GEF proteins together with the comparison of available GEF-GTPase co-structures indicate that the WxxxE-GEF proteins share more than only the common WxxxE-motif [13], [18]. They display several key residues that directly contact the GTPase interface and are important for GEF function. In contrast, alignments of BID-F1 and BID-F2 showed that both domains lack all of these described critical residues besides the central WxxxE/WxxxN motif. Thus, BepF is likely to not represent a WxxxE-family GEF protein. However, the detailed mechanism of how BepF may interfere with Cdc42 and Rac1 signaling remains to be investigated.

We previously showed that BepC and BepF together mediate invasome formation on various cell types [3]. Further, we showed that this process depends on Cdc42, Rac1 and their subsequent downstream signaling partners [2], [3]. With respect to the results presented on this work, the function of BepF in the process of invasome formation is presumably the activation of Cdc42 and Rac1. BepC consists of a FIC domain and a single C-terminal BID domain. Recently, FIC domains have been demonstrated to reversibly modify Rho GTPases by AMPylation, thereby inhibiting their interaction with downstream partners [30]. Thus, it is tempting to speculate that BepC may negatively regulate Cdc42 or Rac1 by AMPylation, thereby contributing to the proposed dynamic activation/inhibition of Cdc42 and Rac1 during the process of invasome formation. However, further work on BepC and the function of its FIC domain is required to answer that question.

In summary, we provide evidence that the *Bartonella* effector protein BepF activates Cdc42 and Rac1 and that this activation functionality is contained in the two BID domains BID-F1 and BID-F2.

## Supporting Information

Figure S1 **In silico analysis of BepF.** (**A**) BepF amino acid sequence. Predicted tyrosine phosphorylation motifs (violet) as well as individual BID domains BID-F1 (red), BID-F2 (green) and BID-F3 (blue). are highlighted. (**B**) NetPhos tyrosine phosphorylation prediction (http://www.cbs.dtu.dk/services/NetPhos/). (**C**) ScanSite tyrosine phosphorylation predictions (http://scansite.mit.edu/).
(TIF)

Figure S2 **Stability test of FLAG- and GFP-tagged fusion constructs.** (**A**) HeLa cells were transfected with indicated plasmids and incubated for 48 h. Following cell lysis, total cell extract was separated by SDS-PAGE, transferred onto a nitrocellulose membrane and probed using anti-GFP antibodies. (**B**) Indicated *Bhe* strains were induced for 48 h on CBA-blood plates and thereafter lysed. Total *Bhe* lysates were separated by SDS-PAGE, transferred onto a nitrocellulose membrane and probed using anti-FLAG antibodies.
(TIF)

Figure S3 **Serines S372 (BID-F1) and S508 (BID-F2) are not essential for BID domain function.** HeLa cells were transfected with indicated plasmids for 24 h and thereafter infected with *Bhe ΔbepA-G*/p*BepC* at an MOI = 500 for 48 h. Following fixation, staining with TRITC-Phalloidin and DAPI and image acquisition by automated epifluorescence microscopy, invasomes were quantified (n>500 cells). Results of at least three independent experiments +/− standard deviation are depicted. Student's t-test was performed as indicated.
(TIF)

Figure S4 **BepF triggers the formation of filopodia-like structures on HeLa cells.** HeLa cells were transfected with indicated plasmids for 48 h. Following fixation, and critical-point drying, cells were visualized by transmission electron microscopy microscopy. Representative images of parallel infections are depicted. Scale bars are indicated.
(TIF)

Figure S5 **BepF triggers the formation of filopodia-like structures on NIH 3T3 cells.** Swiss 3T3 cells were serum-starved for 48 h and thereafter transfected with indicated plasmids for 24 h. Following fixation, and staining with TRITC-phalloidin and DAPI, cells were visualized by confocal microscopy. Representative images of parallel transfections are depicted. Scale bars are indicated.
(TIF)

Table S1 **Bacterial strains and plasmids used in this study.**
(DOC)

Table S2 **Oligonucleotides used in this study.**
(DOC)
