# Supplementary figures and images for: BID-F1 and BID-F2 Domains of Bartonella henselae Effector Protein BepF Trigger Together with BepC the Formation of Invasome Structures
Source: PLoS One. 2011 Oct 17;6(10):e25106. doi: 10.1371/journal.pone.0025106 (PMC3197191; doi:10.1371/journal.pone.0025106)

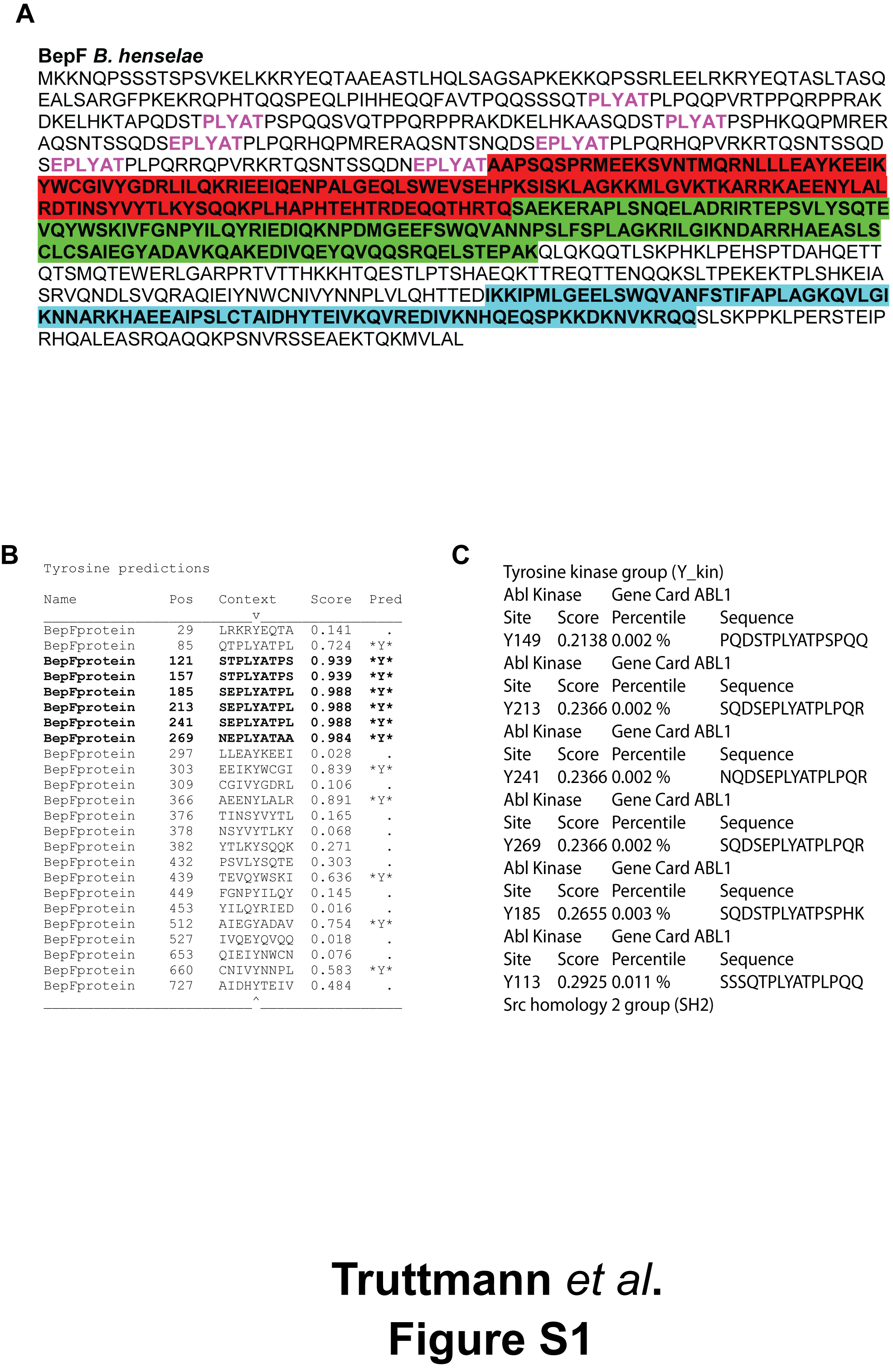

Supplement: Figure S1 — In silico analysis of BepF. (A) BepF amino acid sequence. Predicted tyrosine phosphorylation motifs (violet) as well as individual BID domains BID-F1 (red), BID-F2 (green) and BID-F3 (blue). are highlighted. (B) NetPhos tyrosine phosphorylation prediction (http://www.cbs.dtu.dk/services/NetPhos/). (C) ScanSite tyrosine phosphorylation predictions (http://scansite.mit.edu/). (TIF) [file pone.0025106.s001.tif]

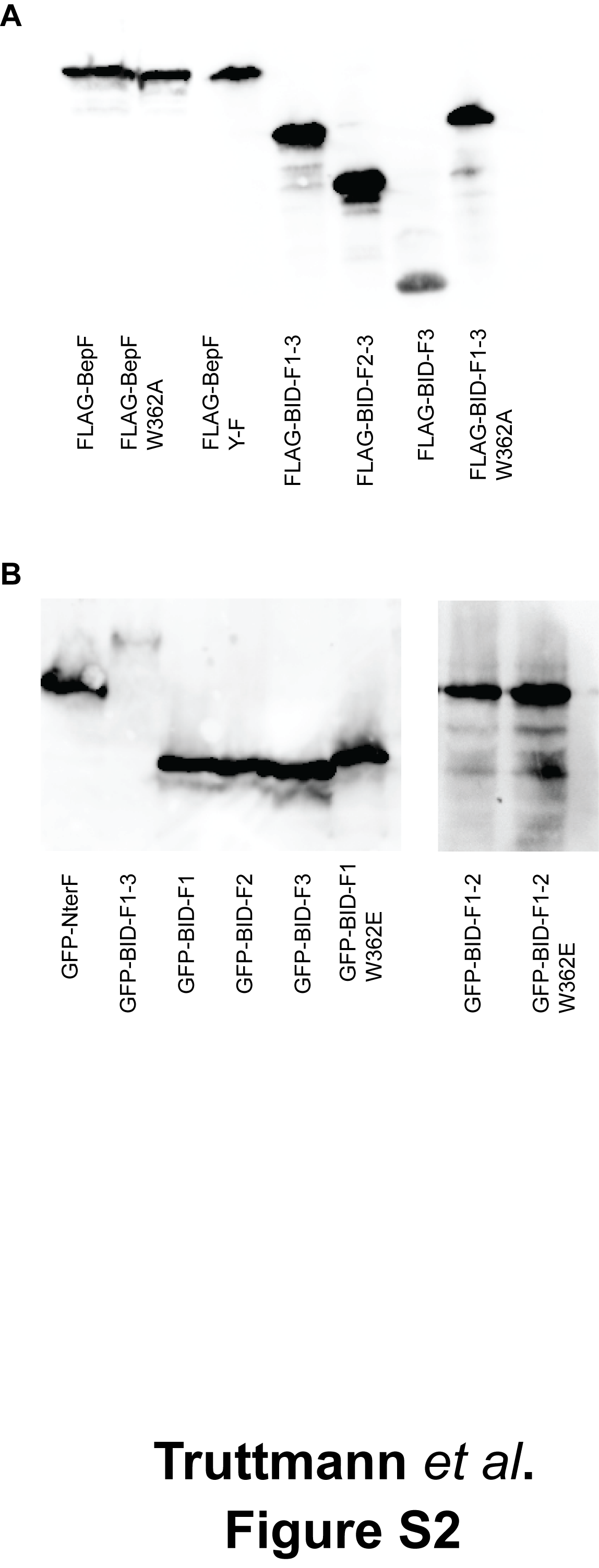

Supplement: Figure S2 — Stability test of FLAG- and GFP-tagged fusion constructs. (A) HeLa cells were transfected with indicated plasmids and incubated for 48 h. Following cell lysis, total cell extract was separated by SDS-PAGE, transferred onto a nitrocellulose membrane and probed using anti-GFP antibodies. (B) Indicated Bhe strains were induced for 48 h on CBA-blood plates and thereafter lysed. Total Bhe lysates were separated by SDS-PAGE, transferred onto a nitrocellulose membrane and probed using anti-FLAG antibodies. (TIF) [file pone.0025106.s002.tif]

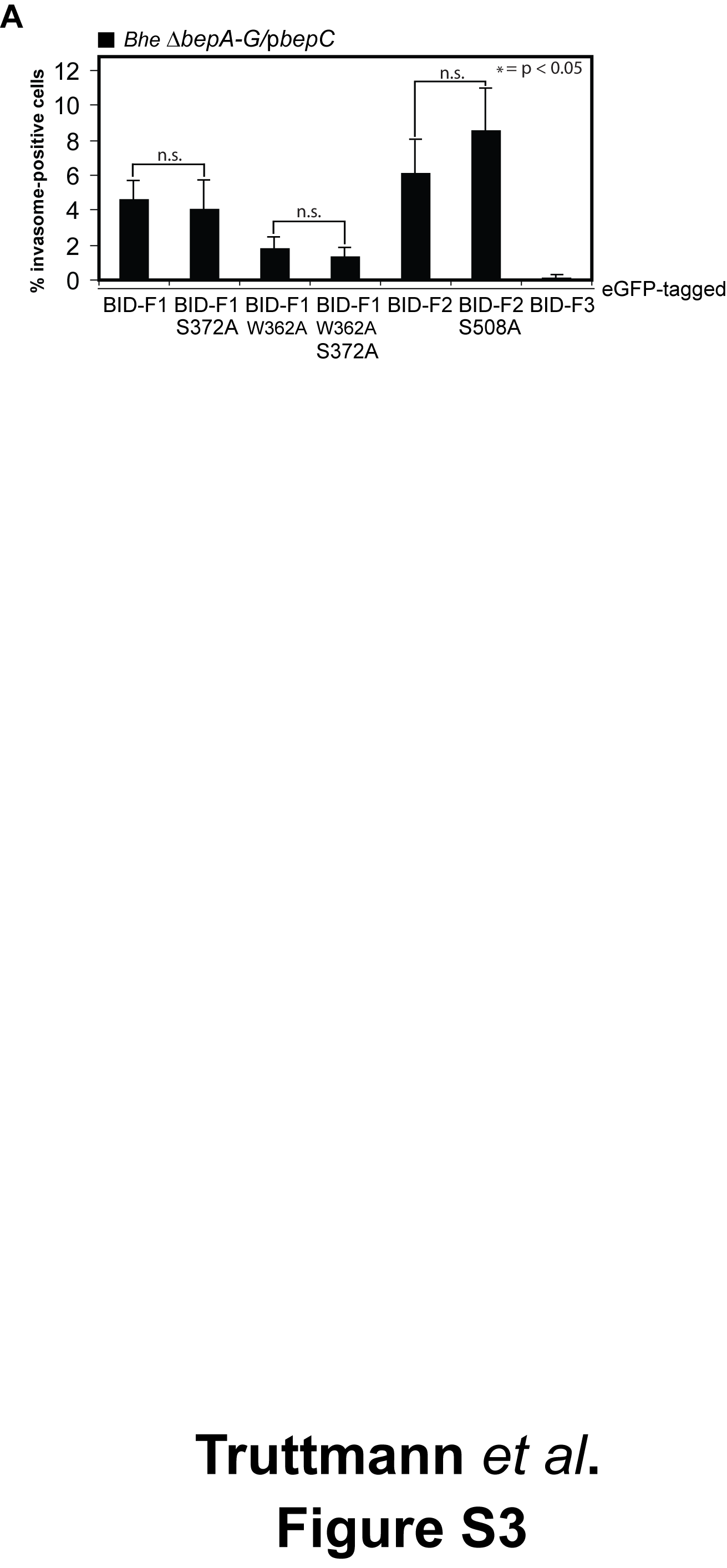

Supplement: Figure S3 — Serines S372 (BID-F1) and S508 (BID-F2) are not essential for BID domain function. HeLa cells were transfected with indicated plasmids for 24 h and thereafter infected with Bhe ΔbepA-G/pBepC at an MOI = 500 for 48 h. Following fixation, staining with TRITC-Phalloidin and DAPI and image acquisition by automated epifluorescence microscopy, invasomes were quantified (n>500 cells). Results of at least three independent experiments +/− standard deviation are depicted. Student's t-test was performed as indicated. (TIF) [file pone.0025106.s003.tif]

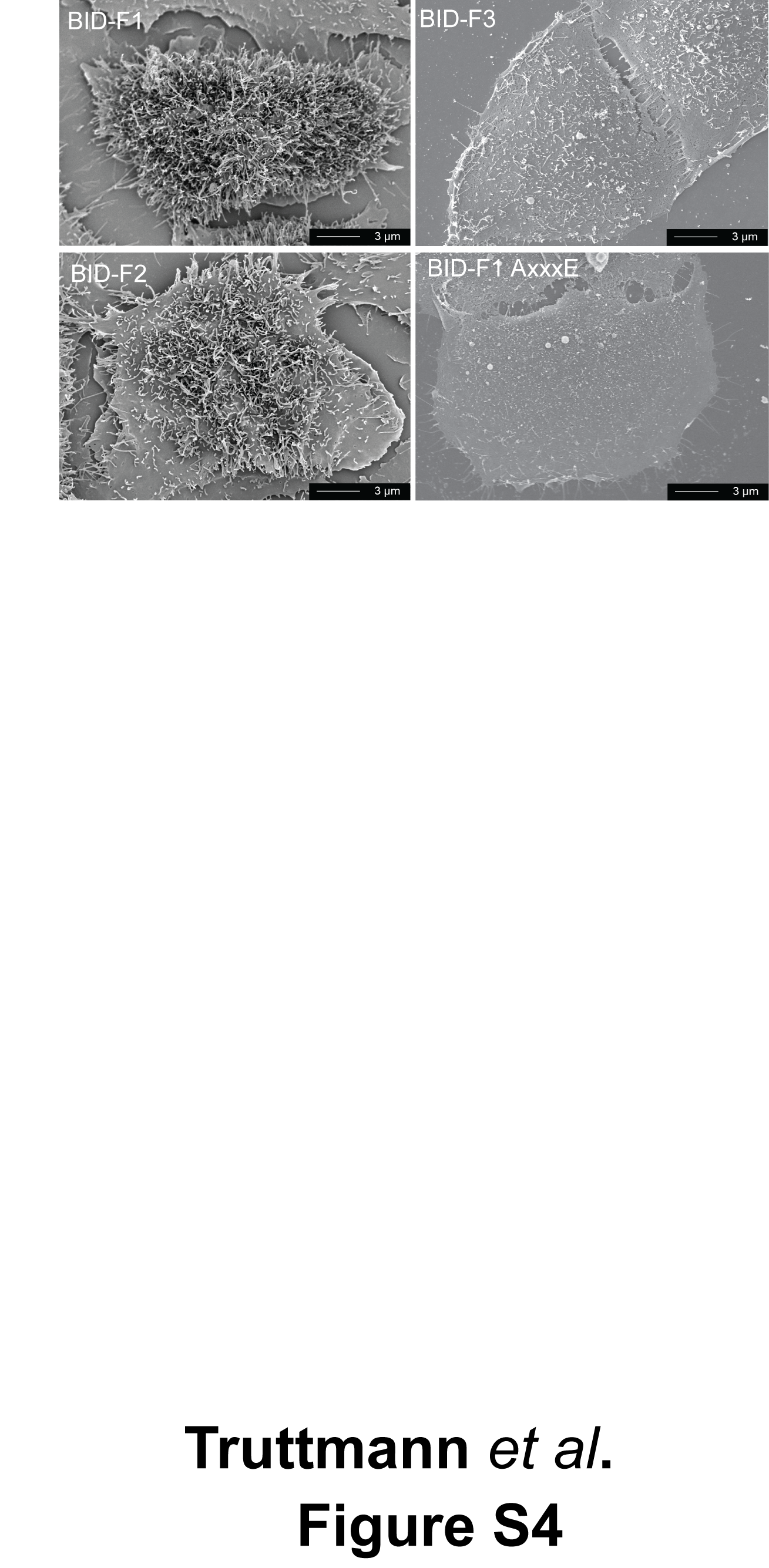

Supplement: Figure S4 — BepF triggers the formation of filopodia-like structures on HeLa cells. HeLa cells were transfected with indicated plasmids for 48 h. Following fixation, and critical-point drying, cells were visualized by transmission electron microscopy microscopy. Representative images of parallel infections are depicted. Scale bars are indicated. (TIF) [file pone.0025106.s004.tif]

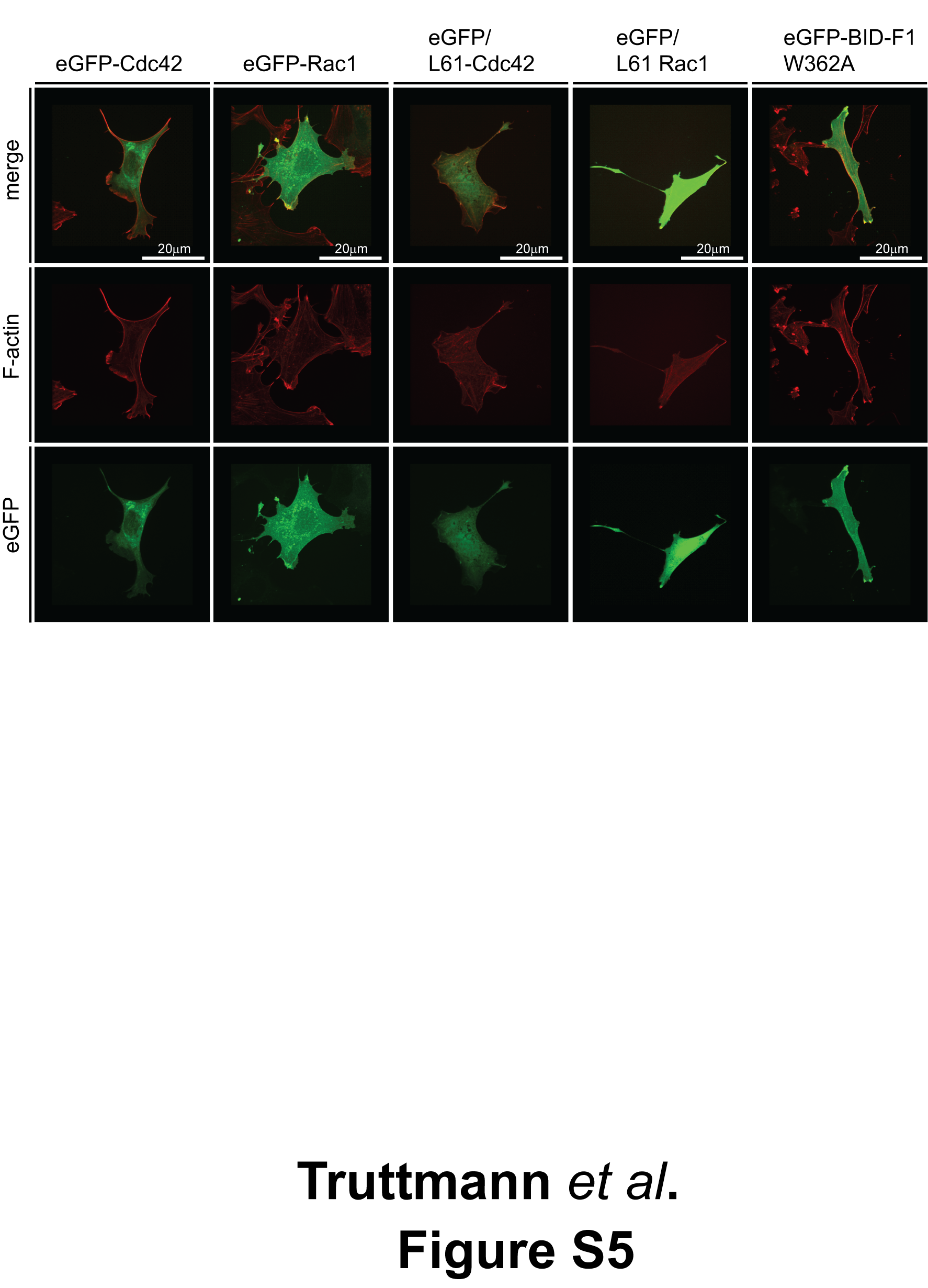

Supplement: Figure S5 — BepF triggers the formation of filopodia-like structures on NIH 3T3 cells. Swiss 3T3 cells were serum-starved for 48 h and thereafter transfected with indicated plasmids for 24 h. Following fixation, and staining with TRITC-phalloidin and DAPI, cells were visualized by confocal microscopy. Representative images of parallel transfections are depicted. Scale bars are indicated. (TIF) [file pone.0025106.s005.tif]
