# Supplementary material for: BID-F1 and BID-F2 Domains of Bartonella henselae Effector Protein BepF Trigger Together with BepC the Formation of Invasome Structures
Source: PLoS One. 2011 Oct 17;6(10):e25106. doi: 10.1371/journal.pone.0025106 (PMC3197191; doi:10.1371/journal.pone.0025106)
Supplement: Table S1 — Bacterial strains and plasmids used in this study. (DOC) [file pone.0025106.s006.doc]

**Table S1:** Bacterial strains and plasmids used in this study

**Strain/Plasmid Genotype or relevant characteristics Reference/Source**

***Bhe* strains**

RSE247 spontaneous Sm^r^ strain of ATCC 49882 ^T^ (Schm*id et a*l., 2004)

MSE150 *ΔbepA-G* mutant of RSE247 (Schule*in et a*l., 2005)

MSE159 MSE150 containing pMS007 (Schm*id et a*l., 2006)

TRB171 MSE150 containing pPG106 (Rhomberg et al. 2009)

TRB169 MSE150 containing pPG107 (Rhomberg et al. 2009)

MTB011 MSE150 containing pMT001 This study

MTB044 MSE150 containing pMT004 This study

MTB041 MSE150 containing pMT005 This study

MTB058 MSE150 containing pMT030 This study

MTB062 MSE150 containing pMT031 This study

MTB076 MSE150 containing pMT052 This study

MTB084 MSE150 containing pMT041 This study

MTB102 MSE150 containing pMT043 This study

***E. coli* strains**

β2150 *F' lacZDM15 lacI^q^ traD36 proA+B+ thrB1004 pro* (Schulein and Dehio, 2002)

*thi strA hsdS lacZ*Δ*M15* Δ*dap*A::erm (Erm^R^) *pir*

NovaBlue *endA1 hsdR1*7(r K12–m K12+) *supE44 thi-1 recA1* Novagen, Madison

*gyrA96 relA1 lac*[*F*’ *proA+B+ lacI^q^*ZΔ*M1*5::T*n10* (Tc^R^)]

BL21 *F–, ompT, hsdSB(rB-, mB-), dcm, gal, λ(DE3)* Novagen, Madison

**Plasmids**

pWay21 Mamalian expression vector for eGFP Molecular Motion lab,

Montana

pCD353 *Bartonella* spp. vector, expressing GFP (Deh*io et a*l., 1998)

peGFP-Cdc42 mammalian expression vector for eGFP-Cdc42 fusion (del Po*zo et a*l., 1999)

peGFP-Rac1 mammalian expression vector for eGFP-Rac1 fusion (del Po*zo et a*l., 1999)

pET15b *E.coli* expression vector Novagen

pMS007 derivative of pPG100, encoding FLAG-BepC (Schm*id et a*l., 2006)

pMT001 derivative of pPG100, encoding FLAG-BepF W362A This study

pMT004 derivative of pPG100, encoding FLAG-BidF1-3 This study

pMT005 derivative of pPG100, encoding FLAG-BepF-YF This study

mutant (113, 149, 185, 213, 241, 269, 297)

pMT030 derivative of pPG100, encoding FLAG-BidF2-F3 This study

pMT031 derivative of pPG100, encoding FLAG-BidF3 This study

pMT041 derivative of pRS79, encoding for BidF3+C-tail BepF This study

pMT043 derivative of pRS79, encoding for BidF1+C-tail BepD This study

pMT052 derivative of pPG100, ecoding FLAG-BidF1-3 This study

W357A

pMT560 derivative of pWAY21, encoding eGFP-BidF1-F3 This study

pMT562 derivative of pWAY21, encoding eGFP-BidF3 This study

pMT563 derivative of pWAY21, encoding eGFP-BepF This study

pMT567 derivative of pWAY21, encoding eGFP-BidF1 This study

pMT591 derivative of pWAY21, encoding eGFP-BidF1-F2 This study

pMT592 derivative of pWAY21, encoding eGFP-BepF aa1-298 This study

pMT593 derivative of pWAY21, encoding eGFP-BidF1 W367A This study

pMT597 derivative of pWAY21, encoding eGFP-BidF1-F2 W367A This study

pMT612 derivative of pWAY21, encoding eGFP-BepF S372A This study

pMT613 derivative of pWAY21, encoding eGFP-BepF This study

W362A, S372A

pMT614 derivative of pWAY21, encoding eGFP-BepF S508A This study

pPG100 *E. coli*- *Bartonella* spp. shuttle vector (Schule*in et a*l., 2005)

pPG106 derivative of pPG100, encoding FLAG-BepF (Rhomberg et al. 2009)

pPG107 derivative of pPG100, encoding FLAG-BepG (Rhomberg et al. 2009)

pRS79 Cre-vector encoding NLS::Cre::BepD (aa 352-534) (Schulein *et al.*, 2005)

pRS110 Cre-vector encoding NLS::Cre::BepF (aa 352-534) (Schulein *et al.*, 2005)

pTR1769 derivative of pWAY21, encoding eGFP-BepC (Rhomberg et al. 2009)
