# Supplementary material for: BID-F1 and BID-F2 Domains of Bartonella henselae Effector Protein BepF Trigger Together with BepC the Formation of Invasome Structures
Source: PLoS One. 2011 Oct 17;6(10):e25106. doi: 10.1371/journal.pone.0025106 (PMC3197191; doi:10.1371/journal.pone.0025106)
Supplement: Table S2 — Oligonucleotides used in this study. (DOC) [file pone.0025106.s007.doc]

**Table S2:** Oligonucleotides used in this study

**Name Sequence^a^ Restriction site construct**

pMT064 AAAAATTCCCCGGGATGACAGCTGCTCCATCACA XmaI GFP-BidF1 / BidF1 W362A

pMT104 GGGAATTCTCTAGATTGCGTTCTGTGTGTTTGTTGTT XbaI GFP-BidF1 / BidF1 W362A

pMT105 ATAATATGCGGCCGCGATGACAGCTGCTCCATCACAATCA NotI HA-GFP-BidF1

pMT106 ATCTTATGGATCCTTGCGTTCTGTGTGTTTGTTGT BamHI HA-GFP-BidF1

pMT128 AAAAATTCCCCGGGATGAGCGCTGAAAAGGAGAGAG XmaI GFP-BidF1-2 / BidF1-2 W362A

pMT129 GGGAATTCTCTAGATTATTTAGCCGGTTCAGTAGAAAG XbaI GFP-BidF1-2 / BidF1-2 W362A

PMT64 AAAAATTCCCCGGGATGACAGCTGCTCCATCACA XmaI GFP-BidF2

pMT130 GGGAATTCTCTAGATTATTTAGCCGGTTCAGTAGAAAG XbaI GFP-BidF2

pMT20 CGCACGCCTCCACAAAGACCACCGCGCGCAAAAGACAAAGA - FLAG-BepF-YF

pMT21 GCGCGGTGGTCTTTGTGGAGGCGTGCGAACTGG - FLAG-BepF-YF

pMT32 GGGAATTCCATATGATGAAAAAAAACCAACCATCC NdeI FLAG-BepF-YF / BepF W362A

pMT31 GGGAATTCCATATGTTAGAGTGCCAG NdeI FLAG-BepF-YF / BidF1-3 /

BidF1-3 W362A / BidF2-3 /

BidF3

pMT67 GGGAATTCTCTAGATTAGAGTGCCAG XbaI GFP-BepF / BidF1-3

pMT68 AATATACCCGGGATGAAAAAAAACCAACCATCCT XmaI GFP-BepF

pMT64 AAAAATTCCCCGGGATGACAGCTGCTCCATCACA XmaI GFP-BidF1-3

pMT62 GACAAACATATGATGCAACAAACACACAGAACGCAAAGCGC NdeI FLAG-BidF2-3

pMT63 GACAAACATATGATGCCCATGCTAGGAGAAGAACTC NdeI FLAG-BidF3

pMT144 TACCCCCCGGGATGAAAAAAAACCAACCATCCT XmaI GFP-NterF

pMT145 CTAGTCTAGATTATGCGTAGAGAGGTTCGTTGTC XbaI GFP-NterF

pMT114 CATACGCGTCGACAAACAGCTGCTCCATCACAATCA SalI NLS-Cre-BIDF1 C-tail BepD

pMT118 CATACGCGTCGACAAGAACTCTCATGGCAAGTTGCAAA SalI NLS-Cre-BIDF3 C-tail BepF

pMT119 TATGTCCCCCGGGTTATTGCTGTCGTTTCACGTTTT XmaI NLS-Cre-BIDF3 C-tail BepF

pMT121 TGATGTCTGTGTTCACCGGGTTGCGTTCTGTGTGTTTGTTG NLS-Cre-BIDF1 C-tail BepD

pMT122 CAACAAACACACAGAACGCAACCCGGTGAACACAGACATCA NLS-Cre-BIDF1 C-tail BepD

pMT123 TATGTCCCCCGGGTTACTCAGTCGAAAGACTGGGCCT XmaI NLS-Cre-BIDF1 C-tail BepD

^a^ Restriction endonuclease cleavage sites are underlined
